# *OsPP65* Negatively Regulates Osmotic and Salt Stress Responses Through Regulating Phytohormone and Raffinose Family Oligosaccharide Metabolic Pathways in Rice

**DOI:** 10.1186/s12284-022-00581-5

**Published:** 2022-07-02

**Authors:** Qing Liu, Jierong Ding, Wenjie Huang, Hang Yu, Shaowen Wu, Wenyan Li, Xingxue Mao, Wenfeng Chen, Junlian Xing, Chen Li, Shijuan Yan

**Affiliations:** 1 Guangdong Key Laboratory of New Technology in Rice Breeding, Guangdong Rice Engineering Laboratory, Rice Research Institute, Guangdong Academy of Agricultural Sciences, Guangzhou, 510640 China; 2 Guangdong Key Laboratory for Crop Germplasm Resources Preservation and Utilization, Agro-Biological Gene Research Center, Guangdong Academy of Agricultural Sciences, Guangzhou, 510640 China

**Keywords:** PP2C, Osmotic stress tolerance, ABA, JA, Raffinose family oligosaccharide, Rice

## Abstract

Although type 2C protein phosphatases (PP2Cs) have been demonstrated to play important roles in regulating plant development and various stress responses, their specific roles in rice abiotic stress tolerance are still largely unknown. In this study, the functions of *OsPP65* in rice osmotic and salt stress tolerance were investigated. Here, we report that *OsPP65* is responsive to multiple stresses and is remarkably induced by osmotic and salt stress treatments. *OsPP65* was highly expressed in rice seedlings and leaves and localized in the nucleus and cytoplasm. *OsPP65* knockout rice plants showed enhanced tolerance to osmotic and salt stresses. Significantly higher induction of genes involved in jasmonic acid (JA) and abscisic acid (ABA) biosynthesis or signaling, as well as higher contents of endogenous JA and ABA, were observed in the *OsPP65* knockout plants compared with the wild-type plants after osmotic stress treatment. Further analysis indicated that JA and ABA function independently in osmotic stress tolerance conferred by loss of *OsPP65*. Moreover, metabolomics analysis revealed higher endogenous levels of galactose and galactinol but a lower content of raffinose in the *OsPP65* knockout plants than in the wild-type plants after osmotic stress treatment. These results together suggest that *OsPP65* negatively regulates osmotic and salt stress tolerance through regulation of the JA and ABA signaling pathways and modulation of the raffinose family oligosaccharide metabolism pathway in rice. *OsPP65* is a promising target for improvement of rice stress tolerance using gene editing.

**Supplementary Information:**

The online version contains supplementary material available at 10.1186/s12284-022-00581-5.

## Background

As sessile organisms, plants are always faced with various environmental stresses such as high salinity, drought, and low temperature (Sharma et al. 2013). These stresses have adverse effects on plant growth and seed production. To survive, plants have developed intricate mechanisms to efficiently perceive external signals and tailor their responses to the precise environmental conditions encountered (Shinozaki et al. 2003; Sharma et al. 2013). Reversible protein phosphorylation mediated by protein kinases and phosphatases is one such adaptive cellular activity that allows plants to sense and transduce a broad range of environmental signals (Luan 2003; Fuchs et al. 2013; Singh et al. 2015). Plant protein phosphatases predominantly belong to three classes of protein serine⁄threonine phosphatases, of which the major class is the type 2C protein phosphatase (PP2C) class (Fuchs et al. 2013).

PP2C-type protein phosphatases, which require Mn^2+^ or Mg^2+^ for their activities, are the largest protein phosphatase family in plants and have been reported to play important roles in signal transduction pathways associated with stress tolerance, innate immunity, stomatal closure, seed dormancy, apical hook development, and plant yield (Schweighofer et al. 2004, 2007; Singh et al. 2016; Lu et al. 2017; Nishimura et al. 2018; Wang et al. 2020; Yu et al. 2020; Chen et al. 2021; Wong et al. 2021). For instance, *PP2C49* negatively regulates Arabidopsis salt tolerance by inhibiting the activity of *AtHKT1;1*, whereas *ZmPP2C55* positively regulates maize drought tolerance (Chu et al. 2021; Zhang et al. 2022). AP2C1, which inactivates the mitogen-activated protein kinases *MPK4* and *MPK6*, regulates plant defense responses to both the necrotrophic pathogen *Botrytis cinerea* and hemibiotrophic pathogen *Pseudomonas syringae* in Arabidopsis (Schweighofer et al. 2007; Shubchynskyy et al. 2017). ABA-HYPERSENSITIVE GERMINATION 1 controls seed dormancy and germination by interacting with DELAY OF GERMINATION 1 in Arabidopsis (Née et al. 2017; Nishimura et al. 2018). A *PP2C-1* allele from wild soybean ZYD7 enhances 100-seed weight by enlarging the size of integument cells and activating a series of genes related to seed traits (Lu et al. 2017).

In rice, 90 PP2C genes have been predicted, but the functions of only few members have been reported (Fujii and Toriyama 2008; Singh et al. 2010; Ni et al. 2019). *OsPP18*, *OsPP2C09*, *OsPP108*, and *OsBIPP2C1* have been demonstrated to be involved in rice tolerance to abiotic stresses, such as drought, osmotic, salt, mannitol, and oxidative stresses (Hu et al. 2006; You et al. 2014; Singh et al. 2015; Miao et al. 2020; Min et al. 2021). The PP2C XA21 BINDING PROTEIN15 negatively regulates XA21-mediated innate immunity in rice (Park et al. 2008), and *OsBIPP2C1/2* was found to significantly enhance resistance to tobacco mosaic virus when expressed in tobacco (Hu et al. 2006, 2009). A recent report indicated that *OsPP95*, which is negatively regulated by the ubiquitin E2 conjugase PHOSPHATE2, positively regulates phosphate homeostasis and remobilization by dephosphorylating the phosphate transporters (PTs) *OsPT2* and *OsPT8* and influencing their trafficking from the endoplasmic reticulum to plasma membrane in rice (Yang et al. 2020). Despite these intriguing results, the specific roles of most of the PP2C genes in rice are still largely unknown.

In our previous study to screen for proteins interacting with OsGF14b that are involved in rice drought response (Liu et al. 2019) through yeast two-hybrid assay, we identified that one PP2C protein, OsPP65, was among the candidate interaction partners. But its specific function in rice stress response has not been reported. In this study, we integrated transgenics, transcriptomics and metabolomics analyses to investigate the biological role of *OsPP65* in rice abiotic stress tolerance. We found that the transcription of *OsPP65* (*LOC_Os04g37660*) is remarkably induced by various stresses and that knockout of *OsPP65* enhances tolerance to osmotic and salt stresses in rice. Further analysis showed that *OsPP65* regulates rice responses to abiotic stresses by regulating the abscisic acid (ABA) and jasmonic acid (JA) signaling pathways independently. Moreover, analysis of the primary metabolomes of the wild-type cultivar *Nipponbare* and *OsPP65* knockout plants indicated that the raffinose family oligosaccharide (RFO) metabolic pathway might play important roles in stress tolerance conferred by loss of *OsPP65* in rice.

## Results

### Expression Profile of OsPP65 Under Different Stress Treatments

**Fig. 1:**
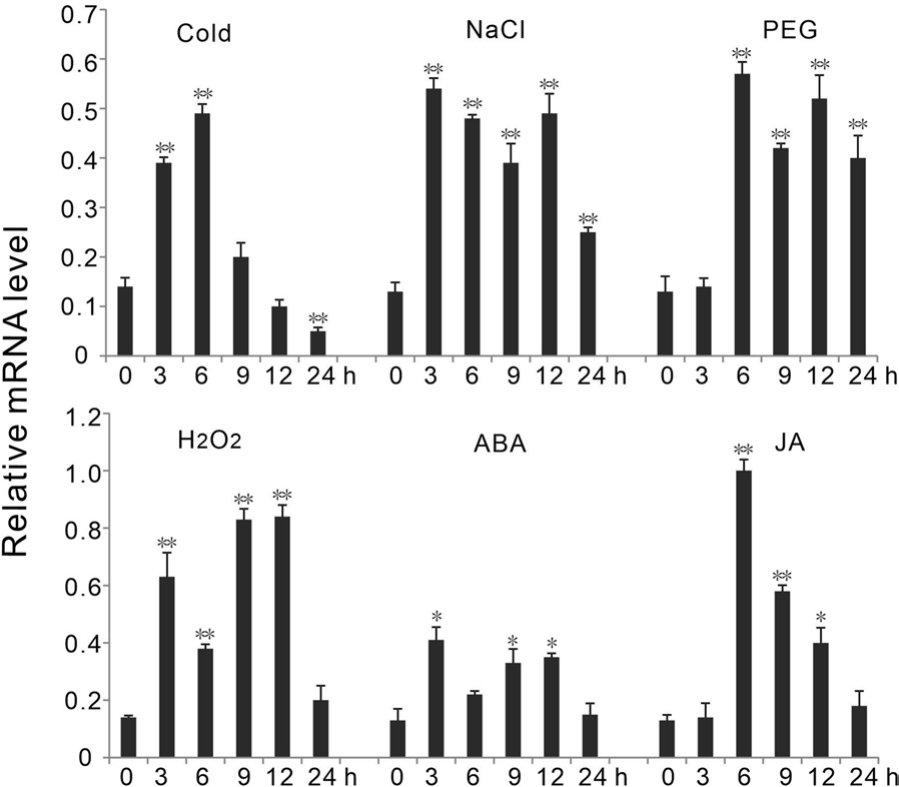
The change in expression of *OsPP65* in response to various stress and chemical treatments. Cold, 8 ℃; NaCl, 150 mM NaCl; PEG, 20% PEG6000; H_2_O_2_, 1% H_2_O_2_, ABA, 100 μM ABA; JA, 100 μM JA. Values represent the means ± SD of three biological replicates (5 plants for each replicate), and “Relative expression level” indicates the expression relative to *EF1α*, which was used as an internal control. The asterisks indicate significant differences compared with the 0 h time point at *P < 0.05 and **P < 0.01 (Dunnett's test)

To analyze the responsiveness of *OsPP65* to various abiotic stresses and hormones in rice, wild-type *Nipponbare* plants were subjected to cold, NaCl, PEG, H_2_O_2_, ABA, or JA treatment, and gene expression analysis was conducted by quantitative RT-PCR (qRT-PCR) at multiple time points from 0 to 24 h post treatment (hpt). The results showed that the transcription level of *OsPP65* was significantly induced at almost all the time points after the NaCl and PEG treatments (Fig. 1). In response to H_2_O_2_ and the hormone treatments, the expression of *OsPP65* first remarkably increased but then decreased to the normal level by 24 hpt. In cold-treated plants, significant induction of *OsPP65* was observed at 3 and 6 hpt, whereas significant reduction of expression was detected at 24 hpt (Fig. 1). These results together indicate that *OsPP65* is a stress-responsive PP2C gene that may play important roles in stress tolerance in rice.

### OsPP65 is Highly Expressed in Rice Seedlings and Leaves and Localized in the Nucleus and Cytoplasm

**Fig. 2:**
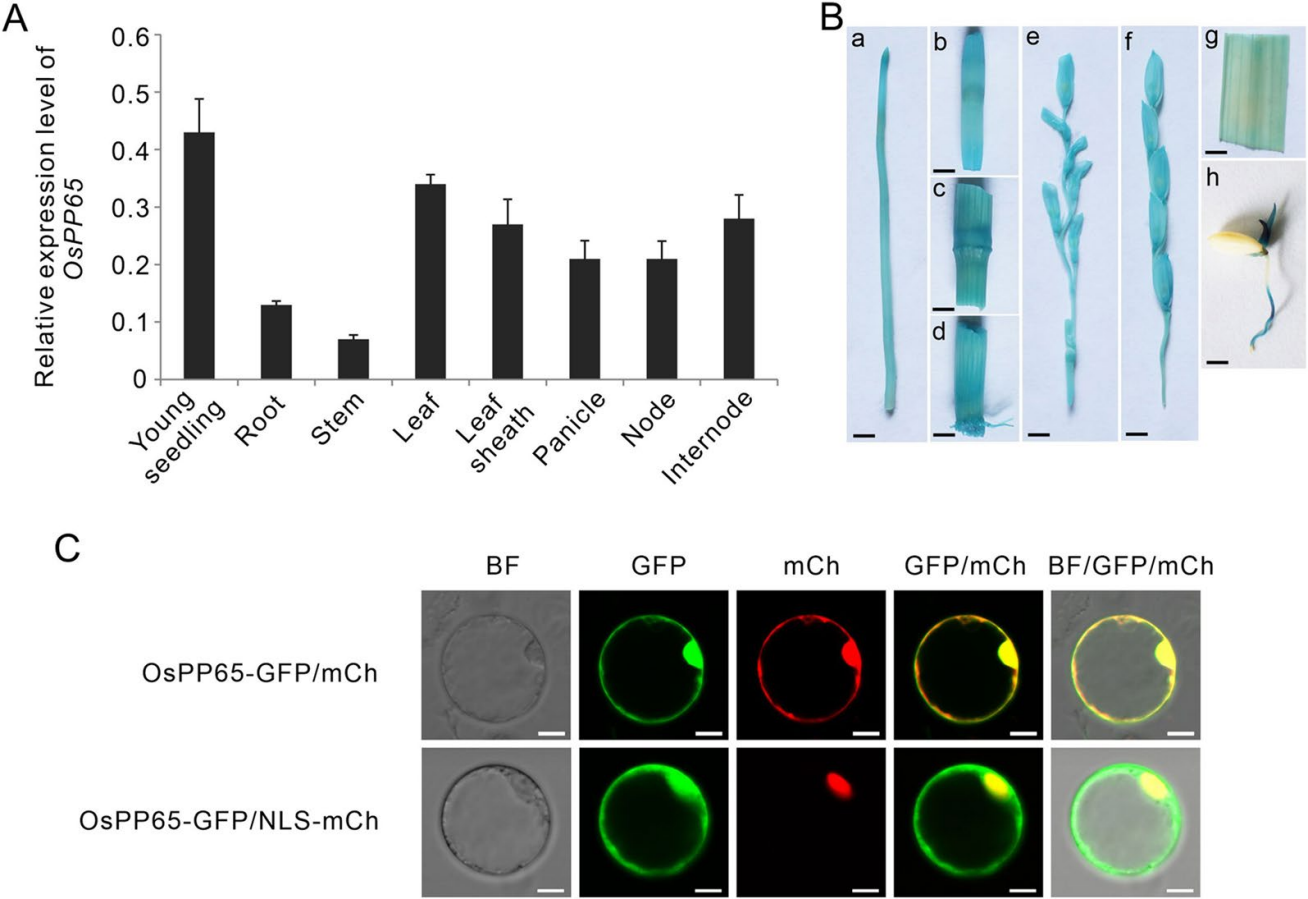
The expression patterns of *OsPP65* in different rice tissues and its subcellular localization. **A** Transcription analysis of *OsPP65* in different rice tissues by quantitative RT-PCR. Values represent the means ± SD of three biological replicates. **B** GUS staining analysis of *OsPP65* promoter-GUS expression in different rice tissues. a, root; b, the third node; c, the second node; d, the first node; e, panicle of the booting stage; f, panicle of the heading stage; g, leaf; h, 3 day-old seedling. Scale bar = 5 mm. C, Subcellular localization of OsPP65 in rice protoplasts. BF indicates bright field, mCh indicates mCherry, and NLS-mCh indicates nuclear localization signal-tagged mCherry. Scale bar = 10 μm

To investigate the biological role of *OsPP65* in rice, we first analyzed the spatial and temporal expression of *OsPP65* in different rice tissues and found that this gene was ubiquitously expressed during the entire rice life cycle, with a relatively higher transcript level in the seedlings and leaves (Fig. 2A). To validate this result, we generated transgenic plants in which expression of *β-glucuronidase* (*GUS*) was driven by the promoter of *OsPP65*. Histochemical analysis revealed GUS activity in all the tissues examined (Fig. 2B), which was consistent with the results of qRT-PCR. The subcellular localization of OsPP65 was determined through transient expression of an OsPP65-GFP fusion protein in rice protoplasts co-expressing a mCherry protein (as a marker for nucleus and cytoplasm localization) or a nuclear localization signal-tagged mCherry protein. At 24 h after transformation, fluorescence microscopy analysis showed that the OsPP65-GFP fusion protein was localized in the nucleus and cytoplasm of rice cells (Fig. 2C).

### Knockout of OsPP65 Enhances Tolerance to Osmotic and Salt Stresses

**Fig. 3:**
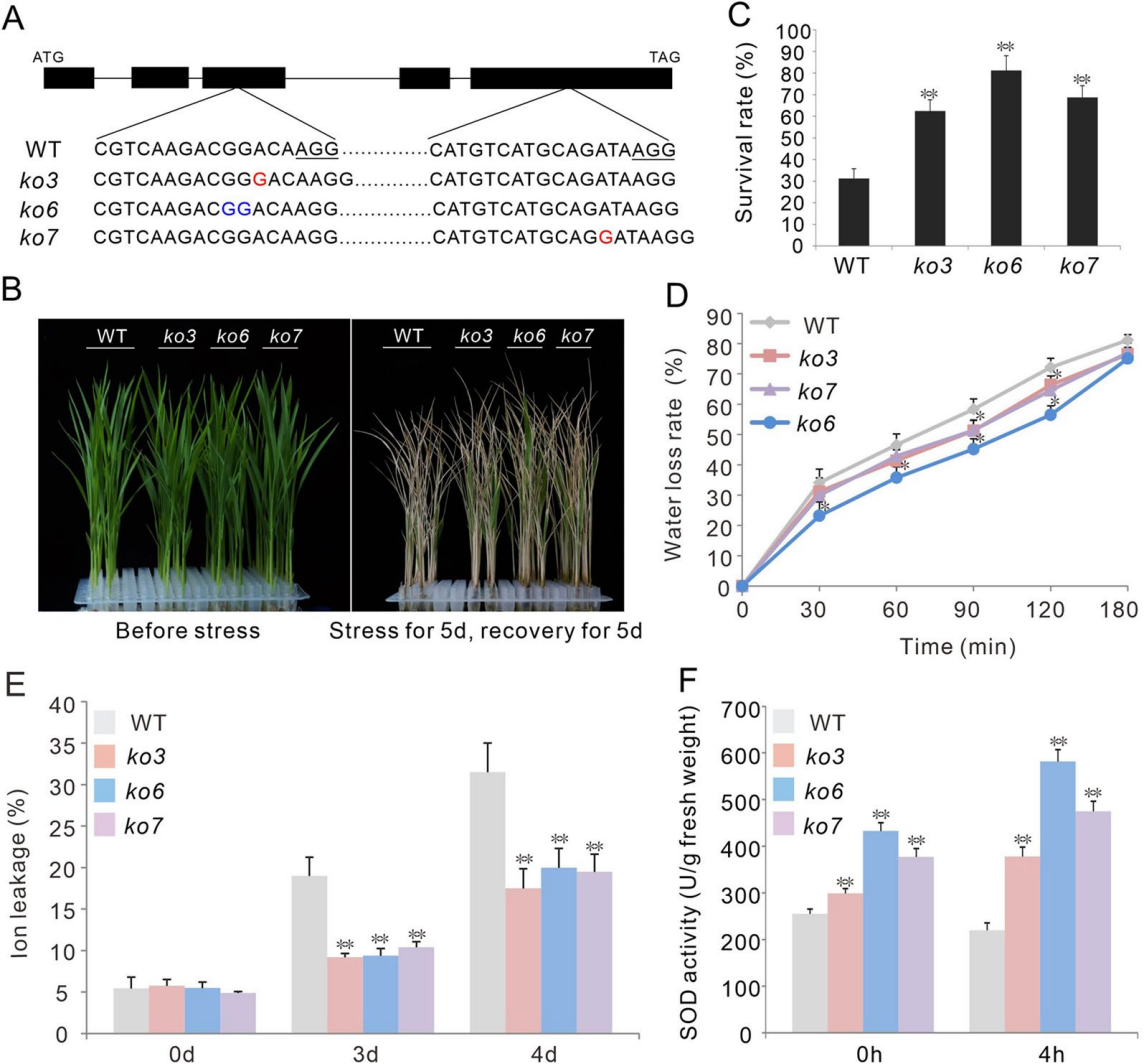
*OsPP65* knockout rice plants show enhanced osmotic stress tolerance. Data represent means ± SD of three biological replicates (16 plants for each replicate), and the asterisks indicate significant differences compared with the wild-type (WT) plants at *P < 0.05 and **P < 0.01 (Dunnett's test). **A** Three homozygous transgenic lines used for phenotype analysis. Inserted nucleotides are shown in red, while missing nucleotides are shown in blue. **B** Phenotypes of the WT and *OsPP65* knockout plants before and after osmotic stress treatment. Scale bars = 5 cm. **C** Survival rates of the osmotic stress-treated plants after 5 days of recovery. D, Relative water loss rate of 2-week-old leaves of WT, *ko3*, *ko6*, and *ko7*. E, Relative ion leakage in rice leaves after osmotic stress for 3 days and 4 days. F, SOD activities in the seedlings of WT and *OsPP65* knockout plants before (0 h) and after (4 h) osmotic stress treatment

To explore the regulatory roles of *OsPP65* in rice responses to abiotic stress, we generated *OsPP65* knockout (*ko*) plants using CRISPR/Cas9 gene editing technology. Two target sites were selected for gene editing and 20 independent transgenic lines were obtained. Through PCR amplification and sequencing analysis, three homozygous lines with different frameshift mutations (*ko3*, *ko6*, and *ko7*) were chosen for stress tolerance evaluation (Fig. 3A, Additional file 1: Fig. S1).

**Fig. 4:**
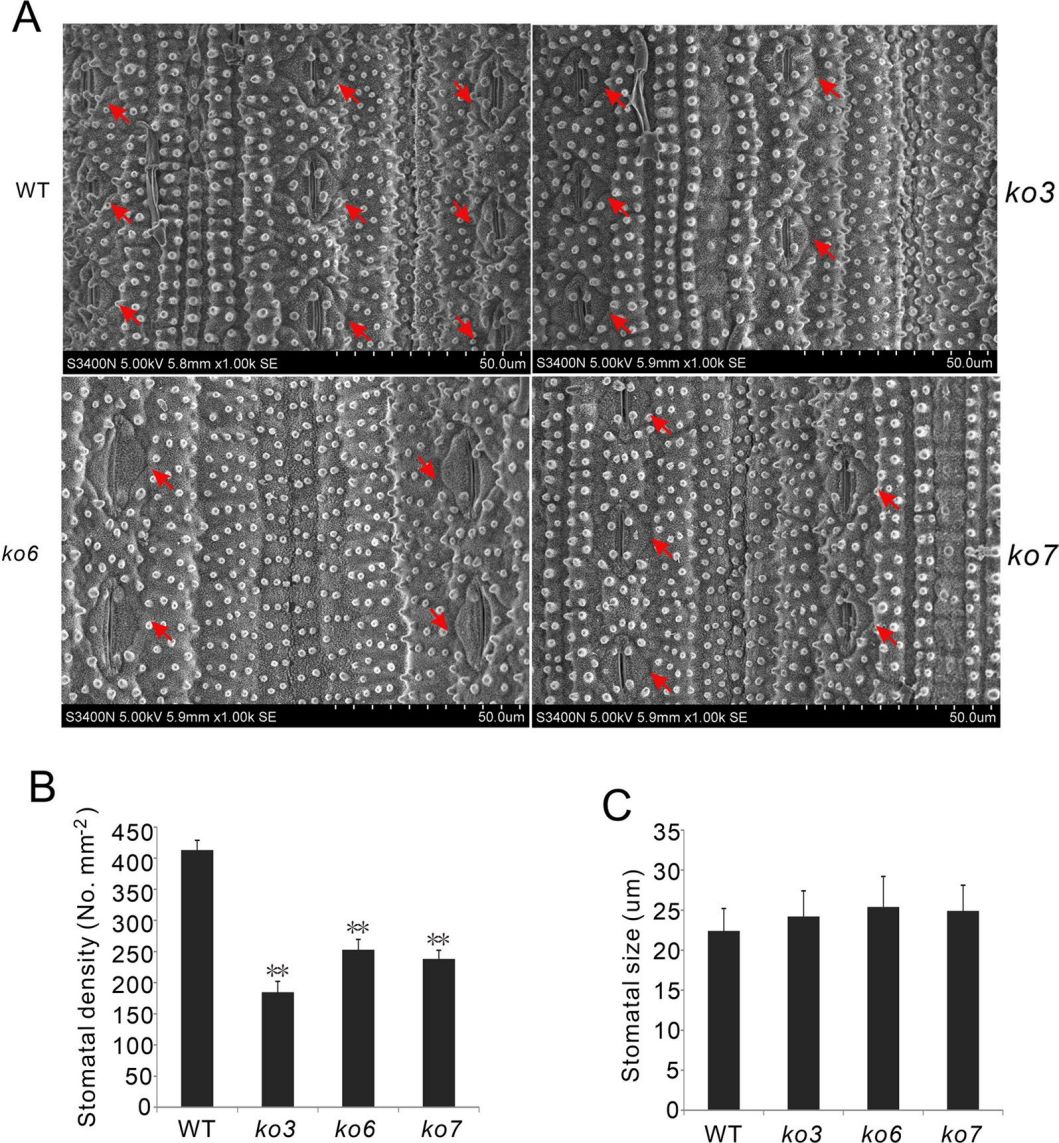
Stomatal density and size in the leaves of WT and *OsPP65* knockout rice plants. **A** Photographs showing the stomatal density in WT and *OsPP65* knockout plant leaves. Red arrows indicate the stomates in leaves. **B** Quantification of the stomatal density in leaves of WT and *OsPP65* knockout plants. **C** Quantification of stomatal size in leaves of WT and *OsPP65* knockout plants. Data are presented as means ± SD of three biological replicates (5 plants for each replicate) and **P < 0.01 (Dunnett’s test)

To assess the role of *OsPP65* in osmotic stress tolerance, 2-week-old rice plants were subjected to 20% PEG 6000 for 5 days and allowed to recover under normal water supply levels (Fig. 3B). After recovery growth for 5 days, the survival rates of the three transgenic lines were all greater than 62%, which was significantly higher than that of the wild-type plants (31%) (*P* < 0.01, Fig. 3C). Ion leakage analysis revealed that the osmotic stress-induced increase in ion leakage was significantly lower in *OsPP65* knockout plants than that in the wild-type plants (*P* < 0.01) (Fig. 3E). Moreover, enzymatic assays showed that the activities of superoxide dismutase (SOD), a reactive oxygen species scavenging enzyme, were much stronger in *OsPP65* knockout plants than in the wild-type plants both before and after PEG treatment (Fig. 3F). To observe the water loss rate in vitro, leaves were detached and exposed to the open air at room temperature and weighed at different times. The water loss rate of the wild-type leaves was higher than that of the leaves of the three *OsPP65* knockout plants at all time points after air-drying (Fig. 3D). Since the water loss rate is closely related to the number and size of stomata, we next quantified the density and size of stomata in leaves. As shown in Fig. 4, the stomata densities were 55%, 39%, and 43% lower in *ko3*, *ko6*, and *ko7*, respectively, as compared with those in the wild-type plants. However, no significant differences in stomata size were observed between the wild-type and transgenic plants (Fig. 4C), indicating that *OsPP65* affects stomata density instead of stomata size in rice. The decrease of stomata density in the *OsPP65* knockout plants is more conductive to maintain the appropriate temperature and water status of leaves than in the wild-type plants when subjected to osmotic stress. Taken together, these results suggest that knockout of *OsPP65* enhances osmotic stress tolerance in rice plants.

To test the function of *OsPP65* in tolerance to salt stress, seeds of the wild-type and *OsPP65* knockout plants were germinated on 1/2 MS medium supplemented with or without NaCl. As shown in Figure S2, no significant differences were identified between the wild-type and *OsPP65* knockout plants in terms of seedling growth in the absence of NaCl. In the presence of 150 mM NaCl, the shoot and root growth of both the wild-type and *OsPP65* knockout plants were severely inhibited at 6 days after germination, but the *OsPP65* knockout plants were markedly less inhibited by NaCl treatment than the wild-type plants (Additional file 1: Fig. S2). Similar results were also observed for 200 mM NaCl, indicating that knockout of *OsPP65* enhances salt tolerance in rice plants.

### OsPP65 Mediated Stress Tolerance is Involved in Activation of the ABA and JA Signaling Pathways

To identify the possible downstream signaling pathways regulated by *OsPP65* in response to stress, RNA sequencing (RNA-seq) analysis of global gene expression was conducted in the wild-type and *ko6* plants under normal conditions and 4 h after osmotic stress treatment. Under normal conditions, only 36 up-regulated genes and 20 down-regulated genes in *ko6* plants were observed to have transcript levels more than twofold higher and lower, respectively, than those in the wild-type plants (false discovery rate ≤ 0.05; Additional file 2: Table S1). However, when subjected to osmotic stress treatment, 139 up-regulated genes and 88 down-regulated genes were identified in *OsPP65* knockout plants compared with wild-type plants. Interestingly, among the 139 up-regulated genes, we found nine genes encoding late embryogenesis abundant (LEA) proteins, which are involved in ABA signaling, implying a role of *OsPP65* in the control of the ABA signaling pathway. In addition, two allene oxide synthase genes (*OsAOS2* and *OsAOS3*), which function in JA biosynthesis, were up-regulatedin *ko6* plants compared with the wild-type plants, suggesting that the stress tolerance conferred by loss of *OsPP65* might also be associated with the JA signaling pathway.

**Fig. 5:**
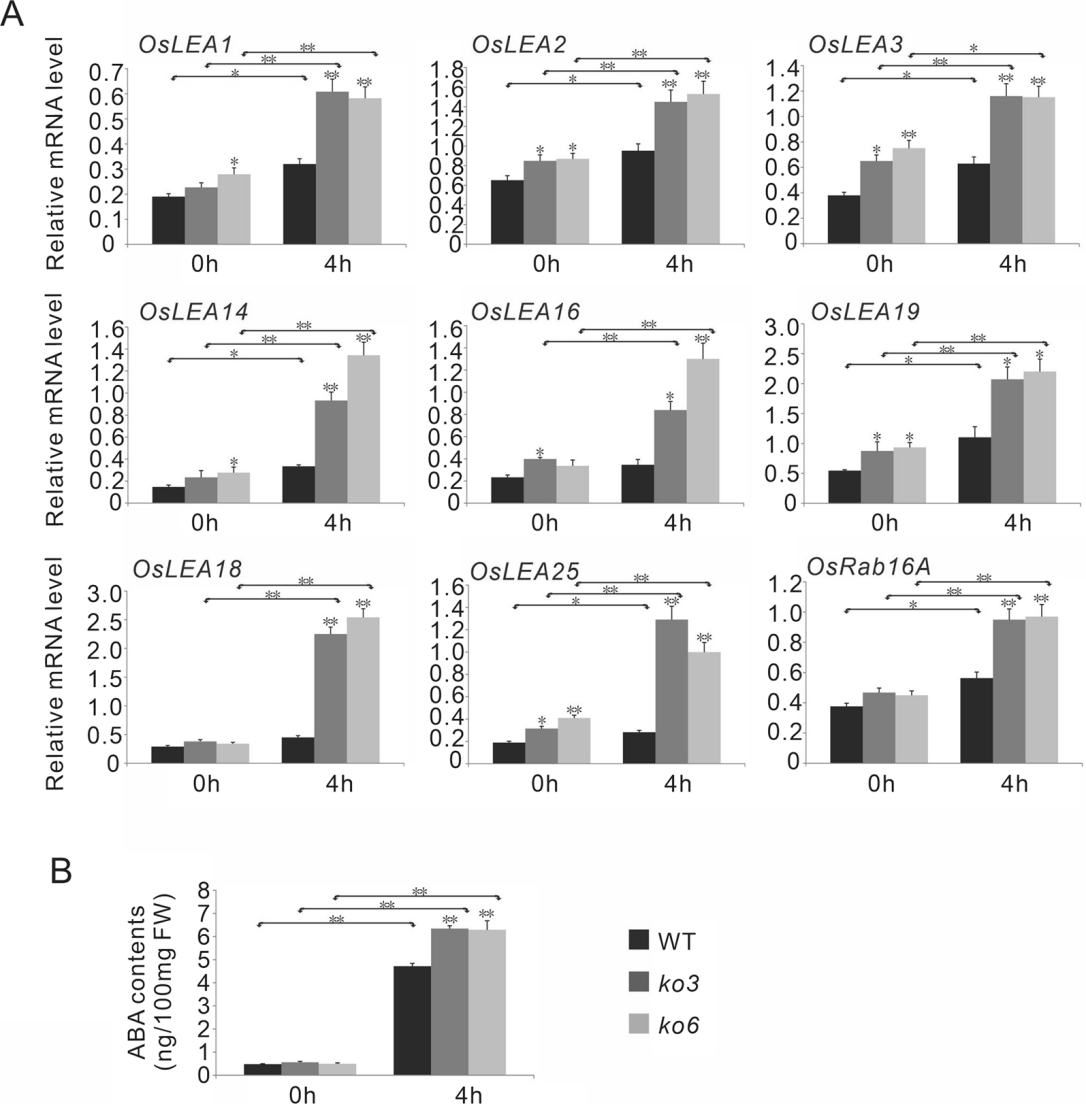
*OsPP65* modulates the expression of genes involved in ABA signaling and the accumulation of ABA in rice. **A** Transcription analysis of the genes involved in ABA signaling in WT and *OsPP65* knockout plants before (0 h) and after (4 h) osmotic stress treatment. **B** Quantification of endogenous ABA levels in the WT and *OsPP65* knockout plants. FW, fresh weight. Data represent means ± SD of three biological replicates (5 plants for each replicate), and the asterisks indicate significant differences compared with the WT plants or 0 h treatment at **P < 0.01 and *P < 0.05 (Dunnett's test)

**Fig. 6:**
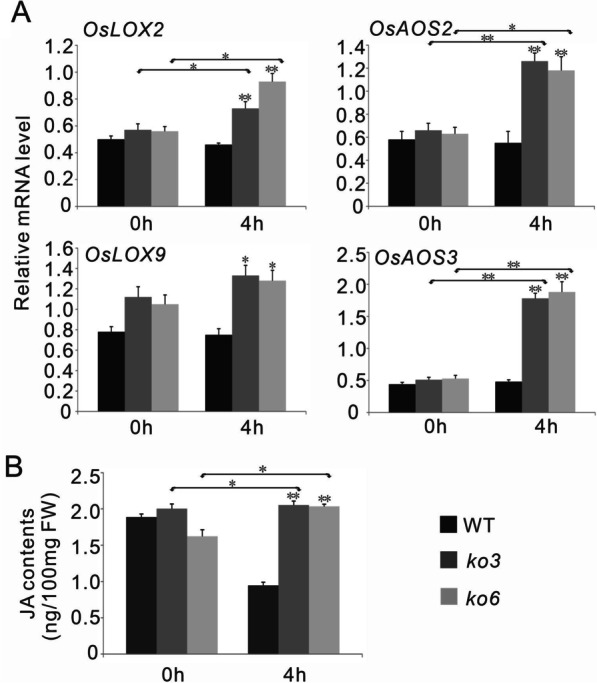
*OsPP65* modulates the expression of genes involved in JA signaling and the accumulation of JA in rice. **A** Expression analysis of genes associated with JA signaling in WT and *OsPP65* knockout plants before (0 h) and after (4 h) osmotic stress treatment. Data represent means ± SD of three biological replicates (5 plants for each replicate). **B** Quantification of endogenous JA levels in the WT and *OsPP65* knockout plants. FW, fresh weight. Data represent means ± SD of three biological replicates (15 plants for each replicate). The asterisks indicate significant differences compared with the WT plants or 0 h treatment at **P < 0.01 and *P < 0.05 (Dunnett's test)

To test these inferences, we first analyzed the expression levels of the differentially expressed genes in wild-type, *ko3*, and *ko6* plants under normal and osmotic stress conditions by qRT-PCR. The transcripts of two lipoxygenase genes (*OsLOX2* and *OsLOX9*), which are involved in JA biosynthesis, were also analyzed in these plants. The results showed that seven *OsLEA* genes (*OsLEA1*/*2*/*3*/*14*/*19*/*25*/*29*) were strongly induced by osmotic stress in both wild-type and *OsPP65* knockout plants, but their expression levels were significantly higher in the *OsPP65* knockout plants than in wild-type plants under osmotic stress conditions (Fig. 6A). Similarly, the transcription levels of *OsLEA16*/*18*, *OsLOX2*, *OsAOS2*, and *OsAOS3* were also remarkably higher in the *OsPP65* knockout plants than in wild-type plants after stress treatment, but induction of their expression by osmotic stress was only observed in the *OsPP65* knockout plants (Figs. 5A, 6A). Consistent with the results of qRT-PCR, the endogenous ABA contents were significantly increased in both wild-type and *OsPP65* knockout plants under osmotic stress, and the ABA concentrations were higher in *OsPP65* knockout plants than in wild-type plants (Fig. 5B). Significant accumulation of JA was also observed in the *OsPP65* knockout plants under osmotic stress conditions, and the JA contents were remarkably higher in the *OsPP65* knockout plants than in the wild-type plants (Fig. 6B). These results indicate that the stress tolerance conferred by loss of *OsPP65* involves activation of the ABA and JA signaling pathways.

To further validate the involvement of ABA and JA signaling pathways in *OsPP65*-mediated stress tolerance, seed germination assays were conducted to analyze the effects of exogenous ABA and methyl jasmonate (MeJA) on the growth of *OsPP65* knockout plants. The results showed that elongation of the roots of *OsPP65* knockout seedlings was less sensitive to exogenous ABA and MeJA treatments than that of the wild-type seedlings (Additional file 1: Figs. S3 and S4). Intriguingly, two ABA-responsive elements and three JA-responsive CGTCA motifs were identified in the promoter of *OsPP65* through bioinformatics analysis (Qin et al. 2020; Additional file 3: Table S2). All these results together demonstrated that stress tolerance conferred by loss of *OsPP65* is at least partially dependent on the activation of the ABA and JA signaling pathways in rice.

The simultaneous accumulation of ABA and JA prompted us to test whether ABA and JA interact synergistically in *OsPP65*-mediated osmotic stress tolerance in rice. Toward this end, we analyzed the transcription of four JA synthesis-related genes (*OsAOS2*, *OsAOS3*, *OsLOX2*, and *OsLOX9*) in the wild-type and *OsPP65* knockout plants after ABA treatment, as well as the transcription of two ABA synthesis-related genes (*OsNCED3* and *OsNCED4*) and five *OsLEA* genes after JA treatment. ABA treatment did not affect the transcription of the four JA synthesis-related genes, and the endogenous contents of JA were not remarkably affected by ABA treatment in both the wild-type and *OsPP65* knockout plants (Additional file 1: Fig. S5). Similar results were also observed for JA treatment (Additional file 1: Fig. S6). These observations suggest that ABA and JA function independently in osmotic stress tolerance conferred by loss of *OsPP65* in rice.

### OsPP65 Mediates Stress Tolerance by Modulating the Metabolism of Raffinose Family Oligosaccharides

**Fig. 7:**
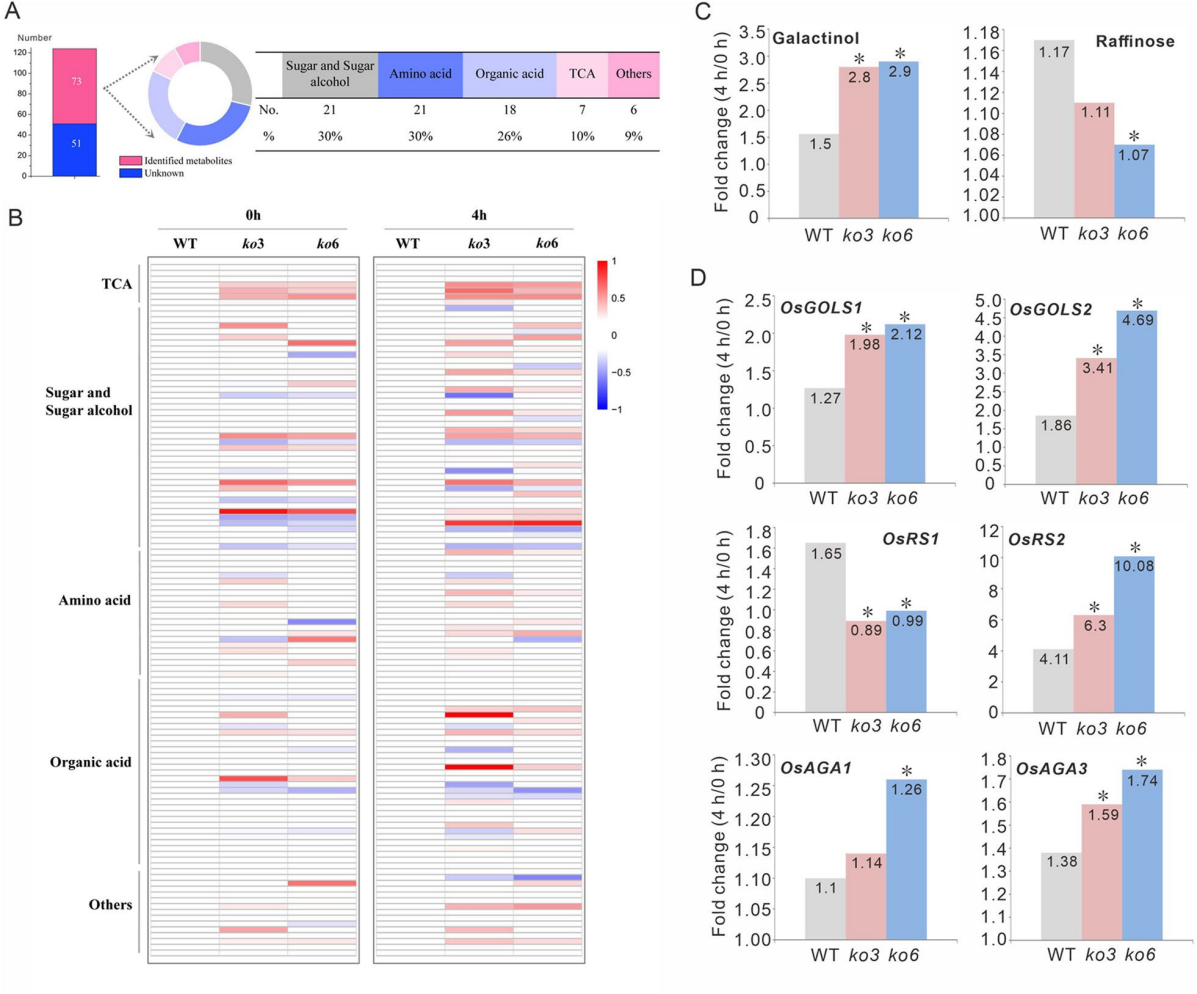
*OsPP65*-mediated stress response involves the regulation of RFO metabolism in rice. **A** The number of primary metabolites and unknown metabolites identified by GC–MS. **B** The number and proportion of identified metabolites in different classes. **C** The fold changes in galactinol and raffinose contents in WT and *OsPP65* knockout plants at 4 h after osmotic stress treatment relative to the 0 h treatment. **D** The fold changes in the expression of key enzymes involved in RFO metabolism in WT and *OsPP65* knockout plants at 4 h after osmotic stress treatment relative to the 0 h treatment. Data represent means ± SD of three biological replicates (15 plants for each replicate), and the asterisks indicate significant differences compared with the WT plants at *P < 0.05 (Dunnett's test)

To further investigate the molecular mechanism underlying the stress tolerance conferred by loss of function of *OsPP65*, primary metabolomic studies were performed using the seedlings of wild-type and *OsPP65* knockout plants (the transgenic lines *ko3* and *ko6*) with (4 h) or without (0 h) osmotic stress treatment by gas chromatography tandem mass spectrometry (GC–MS). The omics data matrix consisted of retention time, an accurate mass-to-charge ratio (*m/z*), and the relative content of each metabolite in each sample (Additional file 4: Table S3). There were 124 metabolite peaks detected in the rice seedlings, and 73 metabolites were conclusively identified based on authentic standards, namely 21 amino acid-related compounds, 18 organic acids, 21 sugar-related metabolites, seven TCA cycle intermediates, and six other compounds (Fig. 7A, Additional file 4: Table S3). To find potential metabolomic variations between wild-type and *OsPP65* knockout plants, orthogonal partial least squares-discriminant analysis was performed, and the results revealed that separation between the wild-type and *OsPP65* knockout plants was more evident at 4 h under osmotic stress (Additional file 1: Fig. S7).

We observed that the magnitudes of the fold changes of most metabolites were higher after 4 h of osmotic stress compared with 0 h both for the wild-type and *OsPP65* knockout plants (Fig. 7B), especially for some TCA cycle intermediates, which were lower in the *OsPP65* knockout lines (Fig. 7B). However, the contents of three metabolites (galactose, galactinol, and raffinose) belonging to the RFO metabolic pathway were higher in both the wild-type and *OsPP65* knockout plants at 4 h after osmotic stress treatment relative to the 0 h treatment (Fig. 7C and Additional file 1: Fig. S8). The fold changes of galactose and galactinol were higher in the *OsPP65* knockout plants than those in the wild-type plants, whereas the fold change of raffinose was lower in the *OsPP65* knockout plants compared with that in the wild-type plants (Fig. 7C). To validate this result, the gene expression of key enzymes involved in RFO metabolism was analyzed. Consistent with the metabolomic results, stress treatment remarkably induced the expression of galactinol synthase 1 and 2 (*OsGOLS1* and *OsGOLS2*), raffinose synthase 2 (*OsRS2*), and alkaline α-galactosidase 1 and 3 (*OsAGA1* and *OsAGA3*) in both the wild-type and *OsPP65* knockout plants, but the fold changes of these enzymes were higher in *OsPP65* knockout plants compared with those in the wild-type plants (Fig. 7D). The higher expression levels of *OsAGA* genes, which function in raffinose catabolism, in the *OsPP65* knockout plants could explain the lower content of raffinose in these plants relative to the wild-type plants. In addition to the products of the RFO metabolic pathway, stress treatment also significantly induced the accumulation of fructose, glucose, glucopyranose, sorbopyranose, rhamnopyranose, and lactulose in both the wild-type and *OsPP65* knockout plants, and the fold changes of these metabolites were remarkably higher in the *OsPP65* knockout plants than those in the wild-type plants (Additional file 1: Fig. S8). These observations together suggest the probably significant roles of these sugars in rice stress response mediated by *OsPP65*.

## Discussion

### OsPP65 Negatively Regulates Osmotic and Salt Stress Tolerance in Rice

PP2C-type protein phosphatases are a group of highly conserved regulatory proteins that are present in virtually all eukaryotic organisms (Singh et al. 2016). Many PP2C proteins have been reported to play important roles in disease resistance, abiotic stress response, and development in plants (Schweighofer et al. 2007; Lu et al. 2017; Nishimura et al. 2018; Wang et al. 2020; Chen et al. 2021). In rice, there are 90 PP2C proteins that are distributed on different chromosomes (Singh et al. 2010). However, only seven of these proteins have been confirmed to function in disease resistance or stress tolerance in previous studies (Hu et al. 2006, 2009; Park et al. 2008; You et al. 2014; Singh et al. 2015; Miao et al. 2020; Yang et al. 2020). The roles of the other members in stress responses are still unknown. In this study, we have demonstrated that *OsPP65* is significantly induced by osmotic and salt stresses. The *OsPP65* knockout rice plants exhibited enhanced tolerance to osmotic stress induced by PEG, as manifested by higher survival rates and SOD activities and lower water loss rates and ion leakage under stress conditions compared with the wild-type plants. The *OsPP65* knockout plants also showed increased tolerance to salt stress at the germination stage, suggesting that *OsPP65* functions as a negative regulator of osmotic and salt stress tolerance in rice. Intriguingly, we also found that *OsPP65* is also involved in leaf blast and bacterial blight resistance in rice (unpublished data). Because of its multiple functions in both abiotic and biotic stresses, we believe that *OsPP65* provides a promising target for improvement of stress tolerance and disease resistance in rice plants using a gene editing approach.

In this study, lower stomata density was identified in the *OsPP65* knockout plants compared with the wild-type plants. *OsSPCH2* and *OsICE1* have been demonstrated to play pivotal roles in determining stomata density in rice. The *osspch2* and *osice1* mutant plants exhibited greatly reduced stomata densities when compared to the wild-type plants (Wu et al. 2019). To analyze that if *OsPP65* could affect the expression of *OsSPCH2* and *OsICE1*, we detected the expression levels of the two genes in wild-type and *OsPP65* knockout plants. The result showed that transcription levels of *OsSPCH2* and *OsICE1* were all down-regulated in the knockout plants compared to the wild-type plants (Additional file 1: Fig. S9), indicating that reduced stomata density conferred by loss of *OsPP65* may at least partially due to the decreased expression of the marker genes of stomata development in rice.

### OsPP65 Mediated Abiotic Stress Response Involves the Regulation of ABA and JA Signaling in Rice

It has been well demonstrated that PP2C proteins are central components of the ABA signaling pathway and function as negative regulators of ABA signaling and responses in plants (Singh et al. 2015, 2016). Phytohormone ABA is a core regulator of plant responses to various abiotic stresses (Shen et al. 2017; Li et al. 2019, 2021; Ullah et al. 2020). Consistent with these previous reports, the gene expression, hormone quantification, and seed germination analyses performed here show that *OsPP65*-mediated abiotic stress response also involves regulation of the ABA signaling pathway in rice. Interestingly, we identified several pieces of evidence showing that there is also a tight association between the *OsPP65* and the JA signaling pathway during rice abiotic stress response. First, the transcripts of four JA synthesis-related genes, *OsLOX2*, *OsLOX9*, *OsAOS2*, and *OsAOS3*, were significantly higher in the *OsPP65* knockout plants compared with wild-type plants after osmotic stress treatment. Second, hormone quantification analysis indicated that the endogenous JA level was significantly higher in the *OsPP65* knockout plants than in wild-type plants under osmotic stress. Third, three JA-responsive elements were identified in the promoter of *OsPP65* and the transcription of *OsPP65* was strongly induced by exogenous JA treatment. Lastly, seed germination experiments demonstrated that the *OsPP65* knockout plants were less sensitive to exogenous JA than the wild-type plants. All these results together suggested that *OsPP65* functions as an negative important regulator of the JA signaling pathway in the regulation of rice response to osmotic stress, consisting with the regulatory roles of JA in rice responses to various abiotic stresses, such as salt, drought and osmotic stresses (Hazman et al. 2015; Kurotani et al. 2015; Wu et al. 2015; Dhakarey et al. 2017; Tang et al. 2020). This result also provides new evidence of the relationship between PP2C proteins and the JA signaling pathway during plant stress response. Interestingly, in plants, the hormone JA is converted into bioactive form JA-isoleucine (JA-Ile) through conjugating isoleucine with JA, and this step is catalyzed by the enzyme *JASMONATE RESISTANT 1* (*JAR1*) (Svyatyna et al. 2014). In the present study, the expression levels of *OsJAR1* were analyzed in both the wild-type and *OsPP65* knockout plants with or without osmotic stress. The results showed that osmotic stress repressed the expression of *OsJAR1* and the expression levels of *OsJAR1* were remarkably lower in the *OsPP65* knockout plants than in the wild-type plants under osmotic stress (Additional file 1: Fig. S10), suggesting that the *OsPP65* knockout plants could lead to less active *OsJAR1* and lastly might resulted in the accumulation of JA when subjected to osmotic stress treatment.

Given that previous studies have indicated that dehydration stress-induced JA accumulation is dependent on ABA in Arabidopsis (Liu et al. 2016a), JA application can enhance foliar ABA concentration in barley (Bandurska et al. 2003), and JA deficiency (*jar1*) diminishes ABA accumulation in Arabidopsis (De Ollas et al., 2015), we performed a set of experiments to test whether the stress tolerance conferred by loss of *OsPP65* involves JA acting in concert with ABA or acting independently. The results showed that ABA biosynthesis was not affected by JA treatment and that JA biosynthesis was not affected by ABA treatment, implying that the JA and ABA signaling pathways function independently in *OsPP65*-mediated abiotic stress response in rice. However, it should be pointed out that the effects of JA on other aspects of the ABA pathway (i.e., distribution, transport, and signaling) and the effects of ABA on other aspects of the JA signaling pathway were not investigated in our present study. Therefore, we cannot rule out the possibility that the JA and ABA signaling pathways act in concert with each other in relation to these other aspects.

### The Important Roles of RFOs in the Osmotic Stress Tolerance Conferred by Loss of OsPP65 in Rice

The functions of galactinol and raffinose in plant abiotic stress tolerance have been well-documented by many studies. For example, rice plants overexpressing *AtGolS2* exhibit improved galactinol content and increased grain yield in terms of panicle number and grain fertility compared with the control plants (Selvaraj et al. 2017). Ectopic expression of *BhGolS1* leads to significant accumulation of galactinol and raffinose as well as elevated drought tolerance in tobacco (Wang et al. 2009). Consistent with previous results, we found that the contents of galactinol and raffinose were increased in both the wild-type and *OsPP65* knockout plants under osmotic stress, and higher galactinol contents were identified in the *OsPP65* knockout plants, which had higher stress tolerance compared with the wild-type plants. However, in contrast to galactinol, the raffinose level was slightly lower in the *OsPP65* knockout plants. We hypothesized that the decreased raffinose level might be due to increased raffinose catabolism in the *OsPP65* knockout plants. Just as expected, the expression levels of *OsAGA1* and *OsAGA3*, two enzymes involved in raffinose catabolism, were remarkably higher in the *OsPP65* knockout plants compared with the wild-type plants after stress treatment. These observations indicate that the rice stress tolerance conferred by *OsPP65* involves in modulating the RFO metabolic pathway. This is the first report to dissect the associations between PP2C protein and the RFO metabolism pathway in rice. Li et al. (2020) revealed that the enhanced drought tolerance conferred by overexpression of *ZmRS* is due to the increased *myo*-inositol levels following galactinol hydrolysis, with the higher ratio of *myo*-inositol to raffinose positively regulating plant drought stress responses. Our results here also suggest that the molecular mechanisms by which RFOs regulate plant abiotic stress tolerance may be different between plant species.

### The Potential Associations Between Sugars and the Phytohormone Signaling Pathways in OsPP65 Mediated Stress Tolerance

In addition to alterations in the RFO metabolic pathway, higher concentrations of fructose, glucose, glucopyranose, sorbopyranose, lactulose, and rhamnopyranose were also observed in the *OsPP65* knockout plants compared with the wild-type plants after osmotic stress, indicating the important roles of these sugars in regulating the abiotic stress response conferred by *OsPP65*. Intriguingly, the associations between sugars and the phytohormone signaling pathways in regulating plant abiotic stresses have been demonstrated by many studies (Rook et al. 2006; Rodriguez et al. 2019; Saddhe et al. 2021). Cheng et al. (2002) reported that exogenous glucose significantly induced the expression of ABA biosynthesis-related genes and thus caused the accumulation of endogenous ABA in Arabidopsis. External application of JA leads to the accumulation of soluble sugars in many crop plants thereby improving the overall plant performance under different abiotic stresses (Ghoulam et al. 2002; Harpreet et al. 2013; Abdelgawad et al. 2014). The simultaneous accumulation of ABA, JA, and sugars observed in the present study prompted us to speculate that there might be cross-talk between these signals in *OsPP65*-mediated osmotic stress tolerance in rice. For instance, the increased levels of ABA and/or JA may lead to the accumulation of sugars, or the increased level of sugars may induce the accumulation of ABA and/or JA in the *OsPP65* knockout plants. Further research will be needed to answer these questions.

## Conclusion

In summary, our results demonstrated that a stress-responsive PP2C protein, OsPP65, negatively regulates osmotic and salt stress tolerance by modulating the ABA/JA signaling pathways and RFO metabolism pathway in rice. *OsPP65* is a promising target for improvement of rice stress tolerance using a gene editing approach.

## Materials and Methods

### Stress Treatments

The japonica cultivar *Nipponbare* was used in the present study. Cold treatment was conducted by incubating 14-day-old rice plants at 8 ℃ in a plant growth chamber. For salt and osmotic stress treatments, 2-week old seedlings were cultured in Kimura B nutrient solution containing 150 mM NaCl and 20% PEG 6000, respectively. For SA, JA, and H_2_O_2_ treatment, 2-week-old rice seedlings were sprayed with a 100 μM hormone solution (SA or JA) or 1% H_2_O_2_. Seedlings were sampled at 3 h, 6 h, 9 h, 12 h, and 24 h after treatment for gene expression analysis.

### Plasmid Construction and Rice Transformation

To generate the vector for gene editing of *OsPP65* using CRISPR/Cas9 technology, two target sites (CGGGTTCGTCAAGACGGACA and CTCACCATGTCATGCAGATA) were selected and the expression cassettes were obtained by overlap PCR using the corresponding primers (*OsPP65*-crispr-6aF/6aR and *OsPP65*-crispr-6bF/6bR, Additional file 5: Table S4). Next, the expression cassettes were inserted into pYLCRISPR/Cas9Pubi-B according to Ma et al. (2015). For promoter analysis, an ~ 2.4-kb fragment upstream of the translational starting site of *OsPP65* was amplified using primers *OsPP65*-GUS-F/R (Additional file 5: Table S4), and this fragment was then inserted into the pCAMBIA1381Z vector. Rice transformation was conducted using the same method as described previously (Toki et al. 2006).

### Quantitative RT-PCR Analysis

Different rice samples which collected from five plants in each biological replicate were ground in liquid nitrogen, and total RNA was extracted using the Trizol reagent (Invitrogen, Carlsbad, CA, USA). Quantitative RT-PCR analysis was performed as described in our previous study (Liu et al. 2016b). Primers used for gene expression analysis are listed in Additional file 5: Table S4.

### Subcellular Localization Analysis

For protein subcellular localization analysis, the coding sequence of *OsPP65* was amplified using primers OsPP65-GFP-F/R (Additional file 5: Table S4) and inserted into the 35S-GFP vector to produce the OsPP65-GFP fusion construct. Subsequently, the fusion plasmids were transiently expressed in rice stem protoplasts or Arabidopsis leaf protoplasts as described previously (Zhang et al. 2011). After incubation in the dark for 24 h at 26 ℃, GFP fluorescence was examined by laser confocal microscopy (Zeiss LSM710, Germany).

### Histochemical GUS Analysis

Different tissues of the transgenic plants transformed with the *OsPP65* promoter-GUS construct were incubated in staining buffer at 37 °C for histochemical GUS analysis as described previously (Liu et al. 2016b). After 24 h in the dark, the tissues were decolored in 75% (v/v) ethanol and photographed by an ordinary Canon camera.

### Determination of Tolerance to Osmotic Stress

For osmotic stress treatment, germinated seeds of the wild-type and *OsPP65* knockout plants were cultured in Kimura B nutrient solution in 96-well plates in a plant growth chamber under 16 h light/8 h dark (26 ℃). The light intensity was 540 μmol m^−2^ s^−1^ and humidity was 75%. After two weeks, the seedlings were subjected to osmotic stress by transferring them into fresh Kimura B nutrient solution containing 20% PEG6000. Five days later, the treated seedlings were recovered in fresh Kimura B nutrient solution for another 5 days. To measure the ion leakage, the second and third leaves were excised from the seedling and incubated in 10 ml of distilled water overnight. The ratio of conductivity values measured by a DDS-11A_T_ conductivity meter (Leici, Shanghai, China) before and after boiling was calculated as the ion leakage from the leaves. The water loss rates of the wild-type *Nipponbare* and *OsPP65* knockout plants were measured using the same method as described by Liu et al. (2014).

For salt stress treatment, seeds of wild-type and *OsPP65* knockout plants were sterilized with 75% ethanol and 2.5% NaClO and germinated on 1/2 MS medium with (150 and 200 mM) or without (0 mM) NaCl for 7 days. Next, the lengths of shoot and primary roots were measured.

### Determination of Stomatal Number

Leaves of 2-week old wild-type *Nipponbare* and *OsPP65* knockout plants were fixed with 2.5% glutaraldehyde, and pictures of stomata were acquired by scanning electron microscopy (S-3400N, Hitachi, Japan) as described previously (You et al. 2013).

### RNA-Seq Analysis

Seedlings (15 plants for each replicate) of the wild-type *Nipponbare* and *OsPP65* knockout plants (line *ko6*) with (4 h) or without (0 h) osmotic stress treatment were collected for RNA-seq analysis. The quality of the RNA-seq raw reads was assessed, and the reads were trimmed by the software trim_galore with the parameters “–stringency 3 –length 36 -e 0.1”. Sequencing reads that passed quality control were mapped onto the *Nipponbare* (MSU7, *Oryza sativa japonica*) reference genome using STAR (2.7.1a) software (Alexander et al. 2013), and an expression matrix was generated using RSEM (v1.3.1) software (Li and Colin 2011). Differential gene expression was calculated using the DESeq2 package in R with a filtering threshold of twofold or greater alteration in expression and an adjusted *P *value less than 0.05. Three biological replicates were performed for each treatment. The raw data has been submitted to NCBI (http://www.ncbi.nlm.nih.gov/) with accession number PRJNA808176.

### Hormone Quantification and Untargeted Metabolomic Analysis

Seedlings (15 plants for each replicate) of the wild-type *Nipponbare* and *OsPP65* knockout plants (lines *ko3* and *ko6*) with (4 h) or without (0 h) osmotic stress treatments were collected for hormone quantification and metabolomic analysis. Hormones were measured using LC–MS while the metabolomic analysis was conducted using GC–MS as described in our previous studies (Liu et al. 2016c, 2018; Yan et al. 2018, 2021). The GC–MS raw data were processed using MassHunter Qualitative Analysis B.06.00 software (Agilent Technologies Inc., Santa Clara, CA, USA) and MassHunter Quantitative Analysis B.07.01 software (Agilent Technologies Inc., Santa Clara, CA, USA). Afterwards, the NIST mass spectral library and an in-house mass spectral database established using authentic standards were used together for metabolite identification.

### Statistical Analysis

Two independent experiments were conducted for stress treatment, hormone quantification and untargeted metabolomic analysis. Three or four biological replicates were performed for each experiment. Three independent experiments were conducted for stress tolerance analysis, stress-related gene expression analysis, and three biological replicates were performed for each experiment. All results are presented as means ± standard derivation (SD) of three biological replicates. Statistically significant difference analysis is conducted by “Dunnett's test” using IBM SPSS statistics software (version 23).

## Supplementary Information

**Additional file 1: Fig. S1**. Phenotypes of the wild-type (WT) and OsPP65 knockout plants at 30 days after flowering under normal conditions. **Fig. S2**. OsPP65 knockout rice plants show enhanced salt stress tolerance at the germination stage. A, Seeds of the WT and OsPP65 knockout plants were germinated on 1/2 MS medium with (150 and 200 mM) or without (0 mM) NaCl for 7 days. B, Shoot lengths of the germinated seeds of WT and OsPP65 knockout plants. C, Root lengths of the germinated seeds of WT and OsPP65 knockout plants. Data represent means ± SD of three biological replicates (20 plants for each replicate), and the asterisks indicate significant differences compared with the WT plants at *P < 0.05 (Dunnett's test). **Fig. S3**. The OsPP65 knockout seeds were less sensitive to exogenous ABA relative to the wild-type seeds. A, Photograph showing the germinated seeds of WT and OsPP65 knockout plants with or without ABA treatment. B, Shoot lengths of the germinated seeds of WT and OsPP65 knockout plants. C, Root lengths of the germinated seeds of WT and OsPP65 knockout plants. Data represent means ± SD of three biological replicates (20 plants for each replicate) and the asterisks indicate significant differences compared to the WT plants at *P < 0.05 (Dunnett's test). **Fig. S4**. The OsPP65 knockout seeds were less sensitive to exogenous JA relative to the WT seeds. A, Phenotypes of the germinated seeds of WT and OsPP65 knockout plants with or without JA treatment. B, Shoot lengths of the germinated seeds of WT and OsPP65 knockout plants. C, Root lengths of the germinated seeds of WT and OsPP65 knockout plants. Data represent means ± SD of three biological replicates (20 plants for each replicate) and the asterisks indicate significant differences compared to the WT plants at *P < 0.05 (Dunnett's test). **Fig. S5**. Exogenous ABA treatment did not affect the JA signaling pathway in OsPP65 knockout plants. A, Transcription analysis of four JA biosynthesis genes in the WT and OsPP65 knockout plants before and after ABA treatment. B, Quantification of endogenous JA in the WT and OsPP65 knockout plants before and after ABA treatment. FW, fresh weight. **Fig. S6**. Exogenous JA treatment did not affect the JA signaling pathway in OsPP65 knockout plants. A, Transcription analysis of two ABA biosynthesis genes and five OsLEA genes in the WT and OsPP65 knockout plants before and after JA treatment. Data represent means ± SD of three biological replicates and the asterisks indicate significant differences compared to the WT plants at **P < 0.01 and *P < 0.05 (Dunnett's test). B, Quantification of endogenous JA in the WT and OsPP65 knockout plants before and after JA treatment. FW, fresh weight. **Fig. S7**. Score plot generated from the GC–MS metabonomics data using the crossvalidated OPLS-DA model. **Fig. S8**. The fold changes of other sugars or amino acids in WT and OsPP65 knockout plants at 4 h osmotic stress treatment relative to 0 h treatment. Data represent means ± SD of three biological replicates and the asterisks indicate significant differences compared to the WT plants at **P < 0.01 and *P < 0.05 (Dunnett's test). **Fig. S9**. Expression levels of OsSPCH2 and OsICE1 in WT and OsPP65 knockout plants under normal condition. Data represent means ± SD of three biological replicates and the asterisks indicate significant differences compared to the WT plants at *P < 0.05 and **P < 0.01 (Dunnett's test). **Fig. S10**. Expression levels of OsJAR1 in WT and OsPP65 knockout plants with or without osmotic stress treatment. Data represent means ± SD of three biological replicates and the asterisks indicate significant differences compared to the WT plants at *P < 0.05 (Dunnett's test).

**Additional file 2: Table S1**. Differentially expressed genes in ko6 plants by RNA-seq analysis before (0 h) and after (4 h) osmotic stress treatment. |log2 Ratio| ≥1 and FDR ≤ 0.05.

**Additional file 3: Table S2**. Cis-elements analysis of the OsPP65 promoter.

**Additional file 4: Table S3**. 120 metabolites identified by GC-MS in WT and the OsPP65 knockout plants. Number after the metabolite represents the isomer.

**Additional file 5: Table S4**. Primers used for vector construction and quantitative RT-PCR analysis.

## Data Availability

The datasets supporting the conclusions of this article are provided within the article and its additional files.

## Abbreviations

PP2C: Type 2C protein phosphatases
ABA: Abscisic acid
JA: Jasmonic acid
PT: Phosphate transporters
Hpt: Hour post treatment
GUS: β-Glucuronidase
*ko*: Knockout
RNA-seq: RNA sequencing
SOD: Superoxide dismutase
*LEA*: Late embryogenesis abundant protein
*AOS*: Allene oxide synthase 2
*LOX2*: Lipoxygenase 2
MeJA: Methyl jasmonate
GC–MS: Gas chromatography tandem mass spectrometry
*GOLS1*: Galactinol synthase 1
*RS2*: Raffinose synthase 2
*AGA1*: Alkaline α-galactosidase 1
RFOs: Raffinose family oligosaccharides
JA-Ile: JA-isoleucine
JAR1: JASMONATE RESISTANT 1
