# Supplementary figures and images for: OsPP65 Negatively Regulates Osmotic and Salt Stress Responses Through Regulating Phytohormone and Raffinose Family Oligosaccharide Metabolic Pathways in Rice
Source: Rice (N Y). 2022 Jul 2;15:34. doi: 10.1186/s12284-022-00581-5 (PMC9250576; doi:10.1186/s12284-022-00581-5)

Figure S1

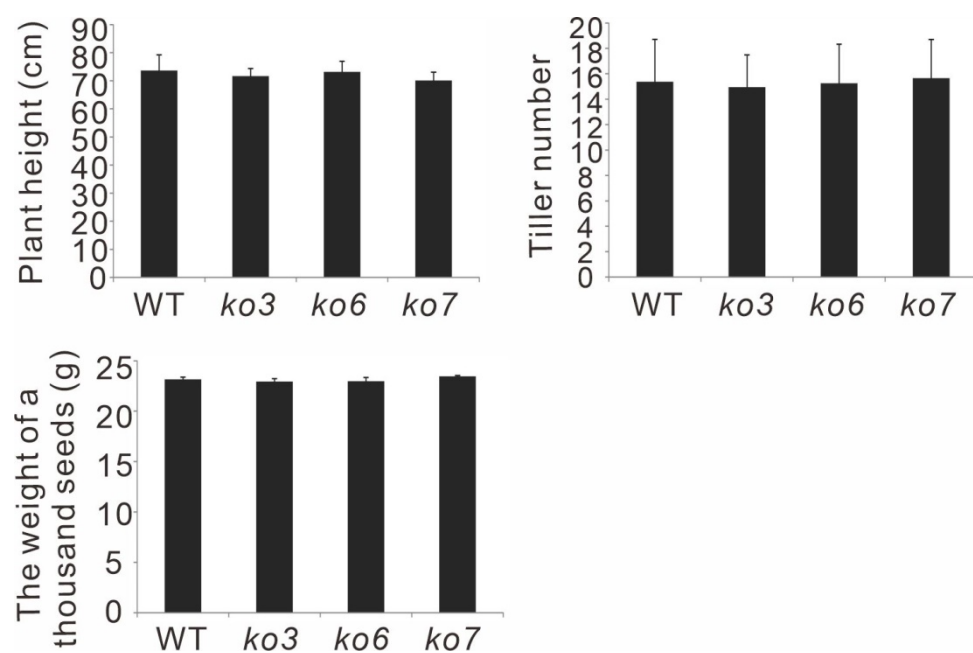

Figure S2

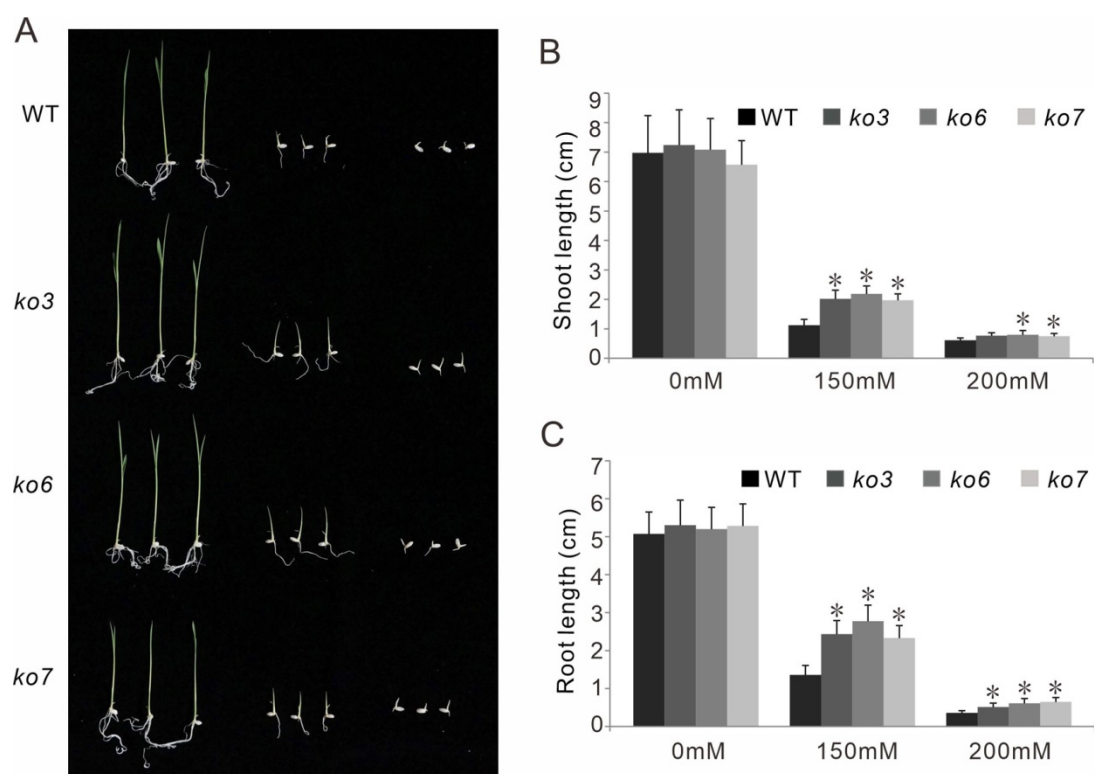

Figure S3

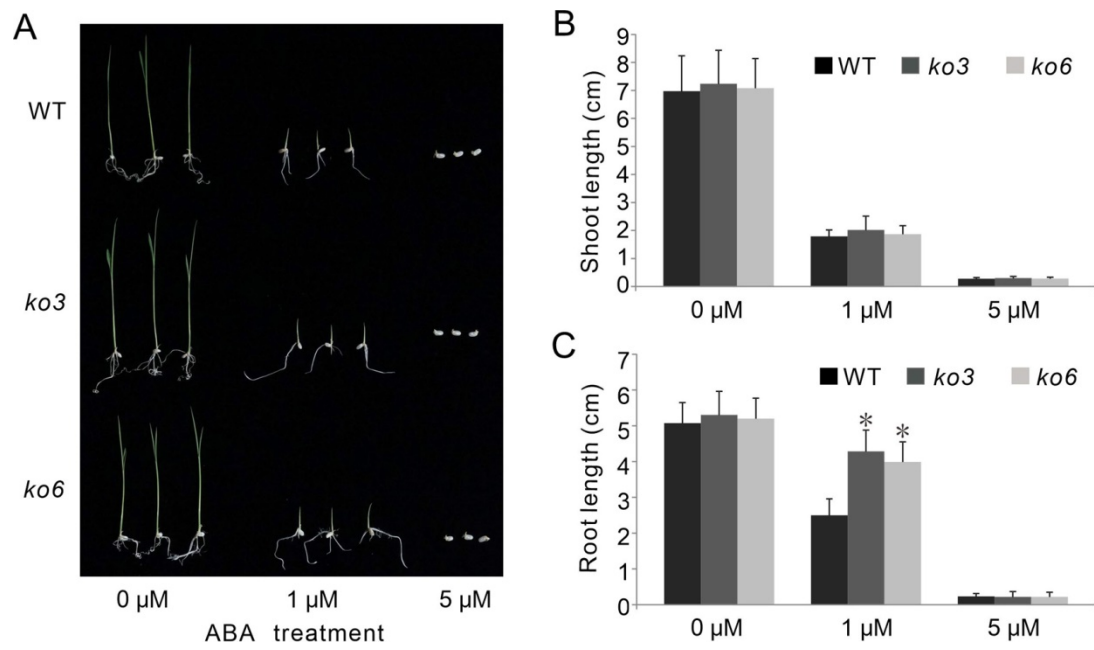

Figure S4

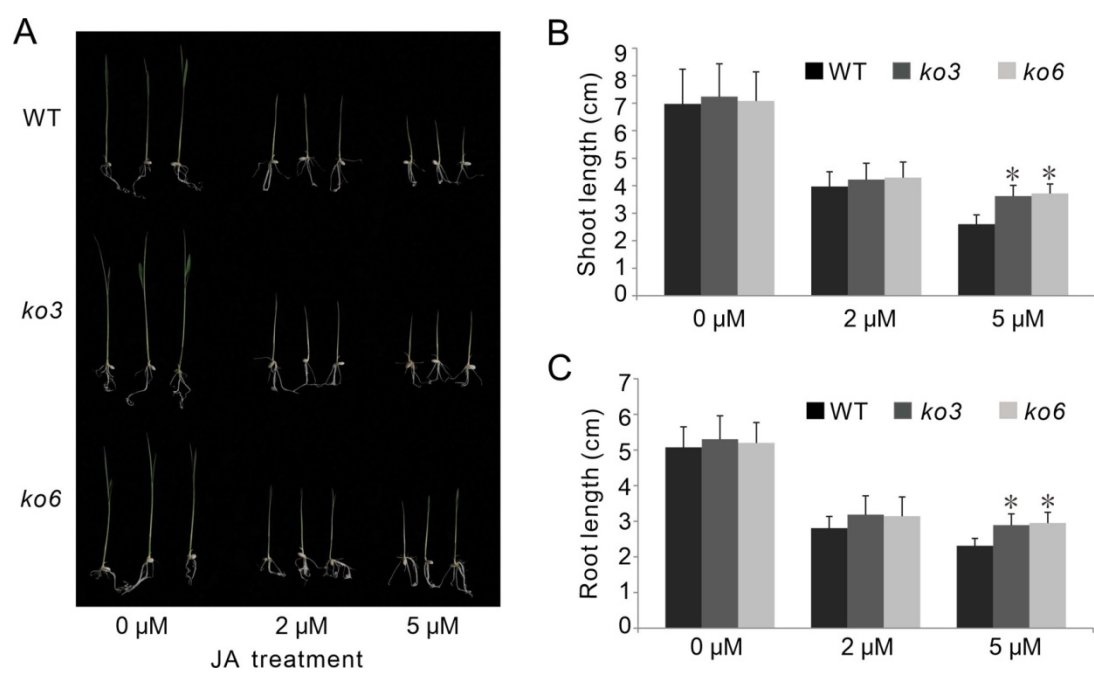

Figure S5

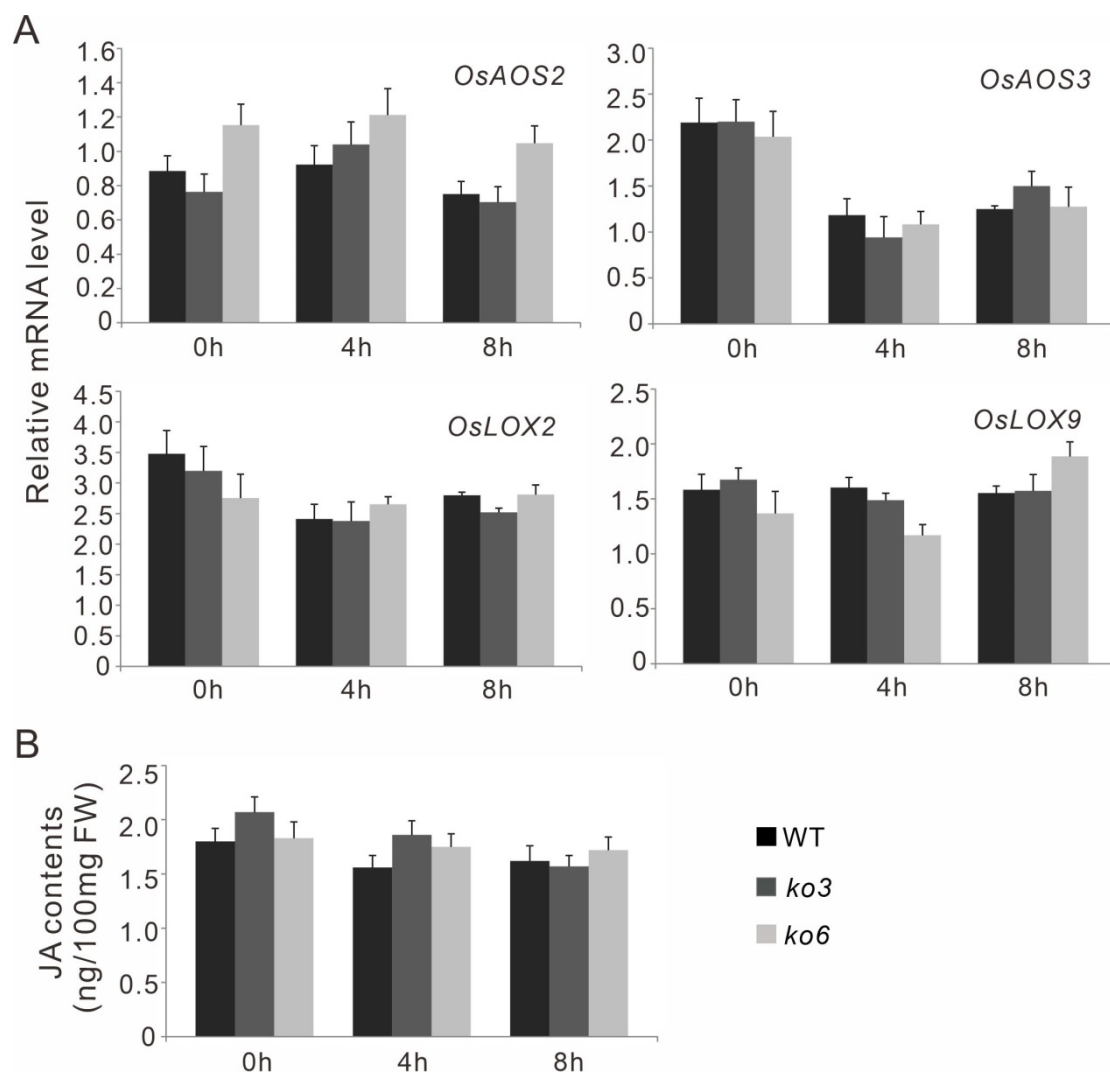

Figure S6

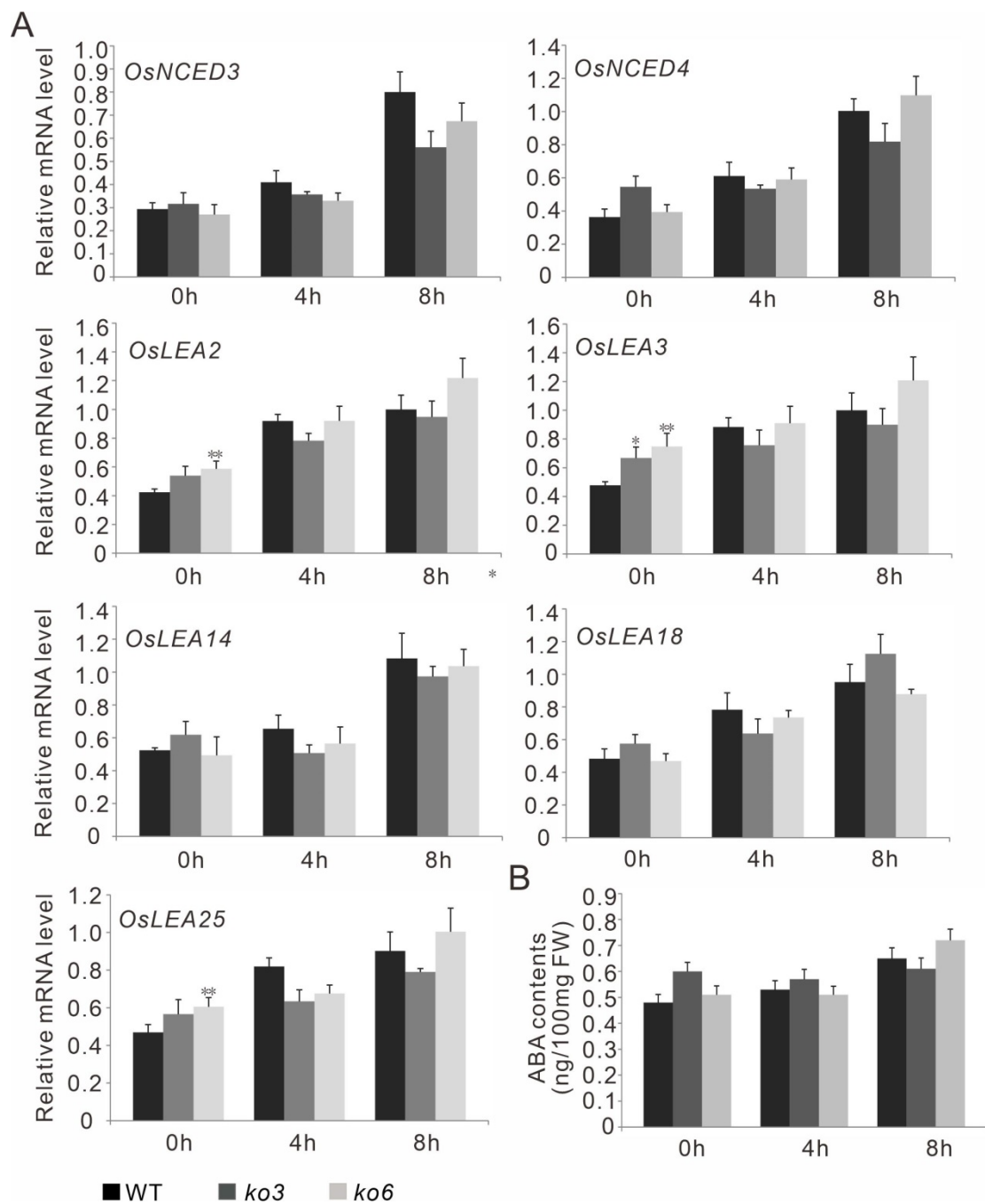

Figure S7

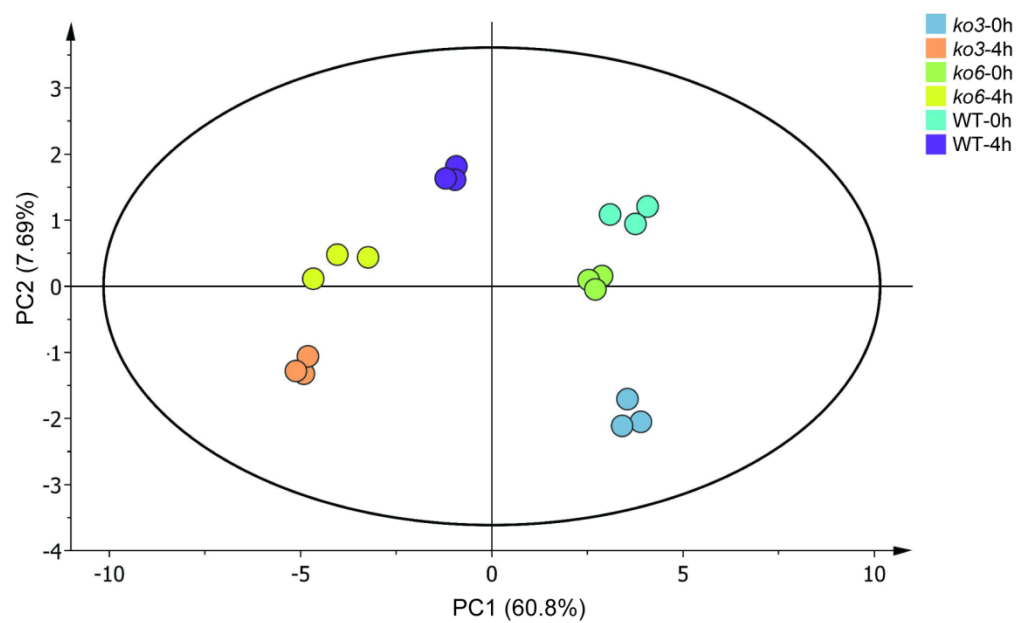

Figure S8

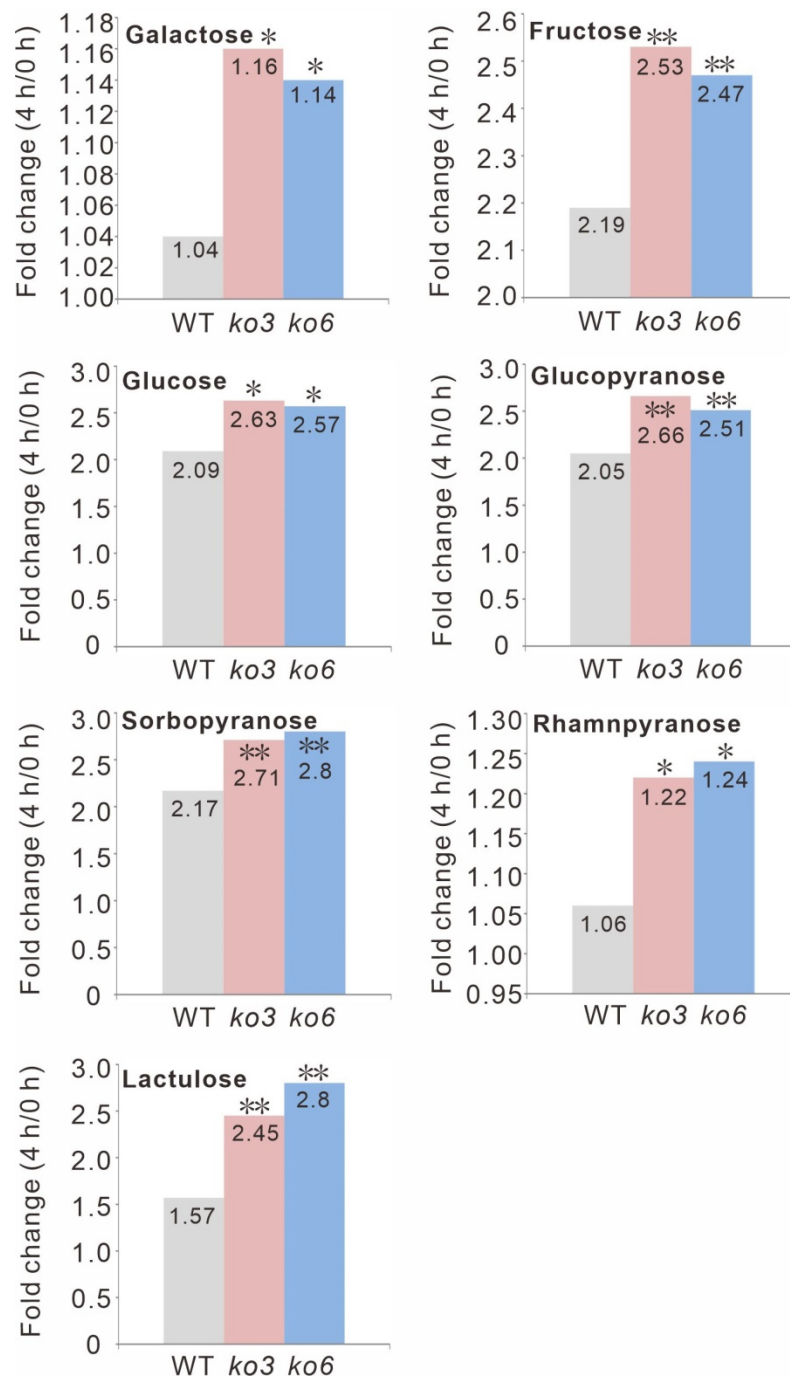

Figure S9

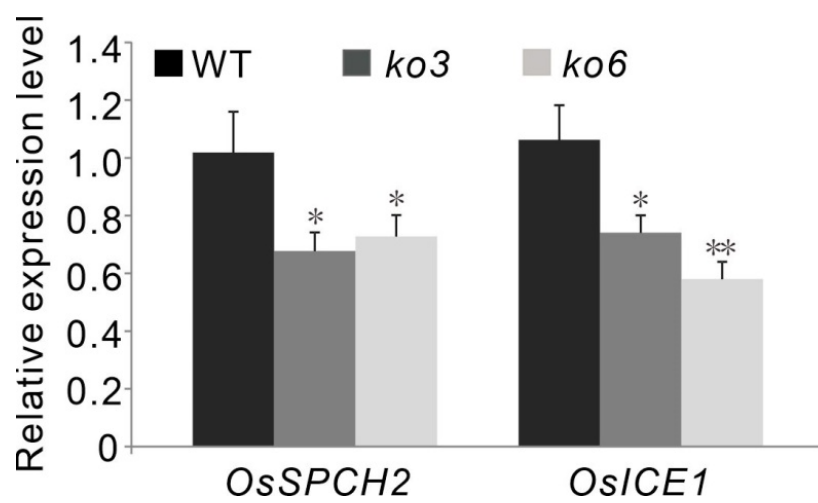

Figure S10

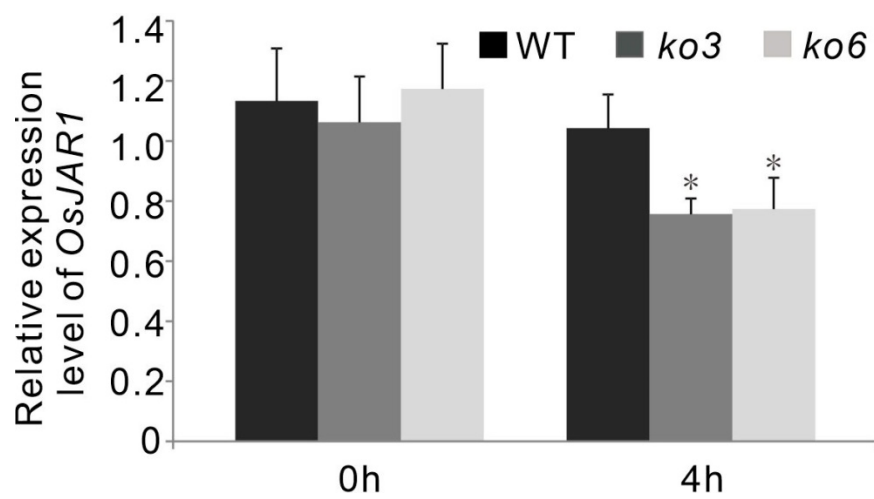

Supplement: Supplementary file 1 — Additional file 1: Fig. S1. Phenotypes of the wild-type (WT) and OsPP65 knockout plants at 30 days after flowering under normal conditions. Fig. S2. OsPP65 knockout rice plants show enhanced salt stress tolerance at the germination stage. A, Seeds of the WT and OsPP65 knockout plants were germinated on 1/2 MS medium with (150 and 200 mM) or without (0 mM) NaCl for 7 days. B, Shoot lengths of the germinated seeds of WT and OsPP65 knockout plants. C, Root lengths of the germinated seeds of WT and OsPP65 knockout plants. Data represent means ± SD of three biological replicates (20 plants for each replicate), and the asterisks indicate significant differences compared with the WT plants at *P < 0.05 (Dunnett's test). Fig. S3. The OsPP65 knockout seeds were less sensitive to exogenous ABA relative to the wild-type seeds. A, Photograph showing the germinated seeds of WT and OsPP65 knockout plants with or without ABA treatment. B, Shoot lengths of the germinated seeds of WT and OsPP65 knockout plants. C, Root lengths of the germinated seeds of WT and OsPP65 knockout plants. Data represent means ± SD of three biological replicates (20 plants for each replicate) and the asterisks indicate significant differences compared to the WT plants at *P < 0.05 (Dunnett's test). Fig. S4. The OsPP65 knockout seeds were less sensitive to exogenous JA relative to the WT seeds. A, Phenotypes of the germinated seeds of WT and OsPP65 knockout plants with or without JA treatment. B, Shoot lengths of the germinated seeds of WT and OsPP65 knockout plants. C, Root lengths of the germinated seeds of WT and OsPP65 knockout plants. Data represent means ± SD of three biological replicates (20 plants for each replicate) and the asterisks indicate significant differences compared to the WT plants at *P < 0.05 (Dunnett's test). Fig. S5. Exogenous ABA treatment did not affect the JA signaling pathway in OsPP65 knockout plants. A, Transcription analysis of four JA biosynthesis genes in the WT and OsPP65 [file 12284_2022_581_MOESM1_ESM.pdf]
